# Differential Diagnosis of Scarlet Fever, Measles and Rubella

**Published:** 1919

**Authors:** 


**The Differential Diagnosis of Scarlet Fever, Measles, and Rubella.** By Lieut.-Col. J. S. Warrack, R. A. M. C. *The British Medical Journal*, November 2, 1918.

It is of great importance that these diseases should be distinguished from each other and from non-infectious diseases in the army, because :

1. The quarantine periods vary;
2. The infectivity varies;
3. The severity varies.

A definite method should therefore be employed in the examination of cases, and the result of the examination accurately recorded.

*The Onset. Scarlet Fever.* Sore throat, shivering, sometimes vomiting, and a rash is seen within 48 hours on the neck, arms, and upper part of the body.

*Measles.* Headache, malaise, coryza the 1st day, Koplick's spots the second day, remaining up to the 5th day. They should be seen 2 days before the rash comes out on the forehead, and still be visible the first 2 days of the rash ; after this period they fade away. The rash comes out late on the 3rd day or early on the 4th.

*Rubella.* Little or no malaise, headache, slight fever, slight sore throat. The rash comes out the 2nd or 3rd day, but in a number of cases the appearance of the rash is the first indication that anything is wrong.

*The Position of the Rash. Scarlet Fever.* The rash avoids the face as a rule. It is seen first on the neck and spreads downwards. The feet are the last to show it. It fades away in the same manner. The rash is likely to be more pronounced on the flexures of the joints.

*Measles.* The rash begins upon the forehead as a blotchy, livid discoloration and spreads downwards. In 24 hours it is out on the legs, and begins to fade on the face by the time it is fully out on the feet and legs. It affects the whole body when it is fully out. It disappears from above downwards.

*Rubella.* This rash may be seen on the face first, but is evanescent. It also spreads from above downwards. The rash may be seen on the dorsum of the feet when it has faded from the body.

*The Character of the Rash. Scarlet Fever.* The rash is composed of fine red points on the background of an erythema ; it may be discrete or confluent. When the latter is the case there is a bright general redness and the skin may be swollen. The flexures may show in addition a hemorrhagic staining of the skin, not necessarily petechial.

*Measles.* Small red papules surrounded by a livid dark blotch. There may be several papules together with one blotch around them. The rash is discrete at first, but rapidly the blotches become larger and the general appearance becomes what is described as measly or mobiliform. The papules may be petechial, but the surrounding blotch is always there.

*Rubella.* 2 types of rash : the scarlatiniform and the coarsely punctate with macules. Two symptoms distinguish it from scarlet fever : the rubella rash is evanescent, and on some parts of the body it is macular. In the scarlatiniform type, the rash is pink, usually found on the trunk and macular on the feet. In the macular type it is composed of large, pink points mixed up with pink macules. The rash is discrete, sometimes petechial as well, always evanescent. It is last seen on the feet and does not become deeper on the flexures. When associated with glandular enlargements and pink eye there should be no difficulty in distinguishing it.

*Early Symptoms.* Sore throat is a prominent feature in scarlet fever, coryza in measles, and slight headache with slight watering of the eyes in rubella. Vomiting is not uncommon is scarlet fever and measles, and shivering in scarlet fever, while in rubella there may be no symptoms at all and the first thing noticeable is rash.

*Glandular Enlargements.* Enlarged glands below the ear are common in scarlet fever and in measles where there is a catarrhal throat and a measles rash on the mucous membranes. In rubella the suboccipitals are not enlarged in some cases, but the posterior cervical is instead.

*State of the Throat.* In all 3 diseases, the involvement of the throat is the exanthem of the disease. In scarlet fever, the throat is bright red with red points, and there may be purulent matter in the nasopharynx; in measles the throat is livid and catarrhal, also swollen with Koplik's spots. In rubella the soft palate only is reddish-pink and the fauces slightly engorged.

*The Tongue.* In scarlet fever the " strawberry " tongue is a feature of the disease. In measles the tongue is livid and flabby, while in rubella the tongue may be furred and flabby.

*The Eyes.* In scarlet fever the eyes are not catarrhal, in measles they are catarrhal, suffused and running. In rubella they are slightly catarrhal and look pink.

*The Significance of Peeling.* Measles does not peel usually, and when it does the peeling is branny and on the body only. Rubella does not peel as a rule. In scarlet fever peeling is the final thing which clinches the diagnosis.

*The Temperature* is raised in all cases. In the first two it is high and remains high. A typical rubella rash may be found with a normal temperature.

*The Possibility of Diagnosis before the Rash Comes Out.* This can be made in all cases of fever and coryza if Koplik's spots are found in the mouth. Fever and sore throat only suggest scarlet fever, if there have been previous cases, but the rash coming out soon settles the diagnosis. Malaise and backache with slight fever and slight coryza suggest rubella.

*The Possibility of Diagnosis with Rash Faded or Fading.* The distribution will be seen, and in scarlet fever there will be staining of the skin, and the bends of the elbows and the shoulders will show dark punctate discolorations. Moreover, peeling will be starting. In measles there will be a blotchy mottling on the parts where the rash has been. In rubella there will be a punctate or macular mottling corresponding to the rash, but this will not last so long.

*Differentiation from Non-Infectious Rashes and Skin Eruptions.* By following the character and distribution of the eruption or rash, the differentiation is extremely easy.

*Mixed Infection.* Diphtheria may complicate scarlet fever and measles. Where there is membrane or sloughing, a throat swab should be taken and serum used without delay.

